# Cryptococcal Neuroradiological Lesions Correlate with Severity during Cryptococcal Meningoencephalitis in HIV-Positive Patients in the HAART Era

**DOI:** 10.1371/journal.pone.0001950

**Published:** 2008-04-16

**Authors:** Caroline Charlier, Françoise Dromer, Christophe Lévêque, Loïc Chartier, Yves-Sébastien Cordoliani, Arnaud Fontanet, Odile Launay, Olivier Lortholary, for the French Cryptococcosis Study Group

**Affiliations:** 1 Centre National de Référence Mycologie et Antifongiques, Unité de Mycologie Moléculaire, CNRS URA 3012, Institut Pasteur, Paris, France; 2 Faculté de Médecine Paris V René Descartes, Hôpital Necker-Enfants Malades, Service des Maladies Infectieuses et Tropicales, Centre d'Infectiologie Necker-Pasteur, Paris, France; 3 Service de Radiodiagnostic, Hôpital du Val de Grâce, Paris, France; 4 Unité de Recherche et d'Expertise en Epidémiologie des Maladies Emergentes, Institut Pasteur, Paris, France; 5 Université Paris-Descartes, Faculté de Médecine, Hôpital Cochin, Pôle de Médecine Interne, CIC de Vaccinologie Cochin-Pasteur, Paris, France; Massachusetts General Hospital, United States of America

## Abstract

Cryptococcal meningoencephalitis has an overall global mortality rate of 20% in AIDS patients despite antifungals. There is a need for additional means of precise assessment of disease severity. We thus studied the radiological brain images available from 62 HIV-positive patients with cryptococcocal meningoencephalitis to analyse the brain lesions associated with cryptococcosis in relationship with disease severity, and the respective diagnostic contribution of magnetic resonance (MR) versus computed tomography (CT).

In this retrospective multicenter analysis, two neuroradiologists blindly reviewed the brain imaging. Prospectively acquired clinical and mycological data were available at baseline and during follow-up. Baseline images were abnormal on 92% of the MR scans contrasting with 53% of the CT scans. MR/CT cryptococcosis-related lesions included mass(es) (21%/9%), dilated perivascular spaces (46%/5%) and pseudocysts (8%/4%). The presence compared to absence of cryptococcosis-related lesions was significantly associated with high serum (78% vs. 42%, p = 0.008) and CSF (81% vs. 50%, p = 0.024) antigen titers, independently of neurological abnormalities. MR detected significantly more cryptococcosis-related lesions than CT for 17 patients who had had both investigations (76% vs. 24%, p = 0.005). In conclusion, MR appears more effective than CT for the evaluation of AIDS-associated cerebral cryptococcosis. Furthermore, brain imaging is an effective tool to assess the initial disease severity in this setting. Given this, we suggest that investigation for cryptococcosis-related lesions is merited, even in the absence of neurological abnormality, if a high fungal burden is suspected on the basis of high serum and/or CSF antigen titers.

## Introduction

*Cryptococcus neoformans* is an encapsulated yeast responsible for severe opportunistic meningoencephalitis mostly in patients with acquired immunodeficiency syndrome (AIDS) [1]–[3]. *C. neoformans* var *grubii* is by far the predominant serotype in HIV-infected patients worldwide. The main presentation is a disseminated meningoencephalitis [1]–[3]. Retrospective radiological studies involving a limited number of HIV-infected patients with cerebral cryptococcosis have been performed in the pre-highly active antiretroviral therapy (HAART) era [4]–[7]. They describe the abnormal cerebral images during cryptococcal meningoencephalitis. The introduction of HAART has significantly modified the radiological presentation of other opportunistic infections [8]. Given this, it is possible that HAART may also have had an impact on the radiological appearances of cerebral cryptococcosis. This is a particularly interesting theory when considering the demonstrated effect of protease inhibitors on some opportunistic pathogens and, specifically, the impact of indinavir or tipranavir on cryptococcal virulence [9]–[10]. Radiological data obtained during the post-HAART era is therefore important, as all the data published so far consists of case reports or small series (n≤4) of HIV-infected patients [11]–[26].

Cryptococcal meningoencephalitis is still associated with an overall 20% mortality rate despite appropriate antifungal therapy, underlining the urgent need for improved management strategies. Most HIV-infected patients with acute neurological symptoms will undergo radiological brain evaluation. However no study has, to date, specifically evaluated the potential utility of neuroimaging to assess the initial severity of AIDS-associated cryptococcosis. Furthermore, analysis of the respective contribution of cranial computed tomography (CT) versus magnetic resonance (MR) in detecting cryptococcal lesions is scarce, in contrast to other opportunistic infections where the beneficial contribution of MR has been clearly established [4], [27].

The aim of the present study was thus to answer these questions using data from a large prospective cohort of HIV-infected patients with culture-proven cryptococcal meningoencephalitis and for whom brain images were available at baseline and during follow-up [3]. In addition, our results were compared with published data on brain imaging during cryptococcosis after a systematic review of the literature.

## Results

### Characteristics of the study population

Sixty two HIV-infected patients were analysed. Of these, 49 (79%) were men, with a median age of 36 years [IQR, 33–44]. Twenty-four (24/62, 39%) were receiving HAART at the time of cryptococcosis diagnosis. Twenty-two (22/62, 36%) patients were reported to have had previous opportunistic infection(s) including cerebral toxoplasmosis (n = 10). Two patients were diagnosed concomitantly with cerebral toxoplasmosis and cryptococcal meningoencephalitis. At baseline, 24 patients had been diagnosed with AIDS for a median of 21 months [IQR, 5–43]. Median viral load was 5.1 log ARN cop/ml [IQR, 4.4–5.5], and median CD4^+^ cell count was 18/mm^3^ [IQR, 7–41]. Twenty seven patients (27/62, 44%) presented with neurological abnormality (ies) at the time of diagnosis, and high serum and cerebrospinal fluid antigen titers (i.e., ≥512) were reported in 34/58 (59%) and 35/56 (63%) of the patients respectively. Serotype A was involved in 49/59 (83%), and serotype D in 10/59 (17%) of the cases.

### Radiological findings at baseline

#### Description of baseline findings

**Figure 1:**
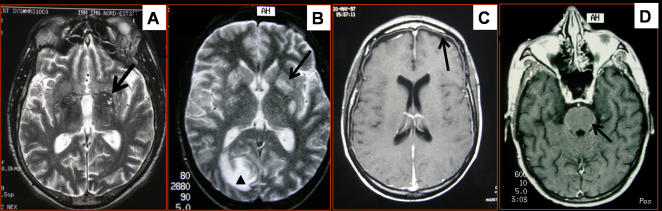
Examples of abnormal radiological findings. A. Magnetic Resonance axial T2-weighted image, displaying bilateral dilated Virchow-Robin spaces (arrow) in the basal ganglia. B. Magnetic Resonance axial T2-weighted image displaying a hyperintense right occipital mass (arrow head) and bilateral dilated Virchow-Robin spaces (arrow). C. Magnetic Resonance axial T1-weighted image with contrast infusion displaying frontal subdural collection (arrow). D. Magnetic Resonance axial T1-weighted image with contrast infusion displaying a basal meningeal enhancement (arrow).

The mean interval between onset of symptoms and initial neuroimaging was 24 days (range 0–104 days). At baseline, 45 patients had single imaging (38 CT, 7 MR), and 17 patients had dual exploration. Baseline CT and MR were normal in 26/55 (47%) and 2/24 (8%) of the patients, respectively (Table 1). Cryptococcosis-related lesions found on CT were rare (13/55, 24%) and, in decreasing order of frequency, consisted of: intracerebral mass(es) (5/55, 9%), dilated VR spaces (3/55, 5%), pseudocysts, hydrocephalus (not attributable to other opportunistic infections), radiological meningitis and edema (2/55, 4% each). By contrast, cryptococcosis-related lesions were observed by MR in 19/24 (79%) cases and consisted of dilated VR spaces (11/24, 46%, Figure 1A), masses (5/24, 21%, Figure 1B), pseudocysts and meningitis (including one case of meningitis with subdural empyema) (2/24, 8% each, Figures 1C, 1D). Nodules or masses (5–40 mm in diameter) were located in the frontal, parietal, occipital gyri, the basal ganglia or *corpus callosum*. Some lesions were enhanced after contrast medium injection, and perilesional edema was observed in one patient with both procedures. One patient with an enhancing mass had possible concomitant cerebral toxoplasmosis.

**Table 1:** Neuroradiological analysis of 55 computed tomographies and 24 magnetic resonance images collected at baseline from 62 HIV-infected patients with culture-proven cryptococcal meningoencephalitis.

Result of brain imaging	Computed tomography (n = 55)	Magnetic resonance (n = 24)
Normal imaging	26 (47%)	2 (8%)
Cryptococcosis-related lesions	13 (24%)	19 (79%)
VR dilatation	3	11
Pseudocysts	2	2
Intracerebral mass(es) [unique/multiple]	5 [3/2]	5 [3/2]
Hydrocephalus	2	-
Radiological meningitis	2^a^	2
Other lesions^b^	20 (36%)	11 (45.8%)
HIV-encephalopathy	5	6
Cerebral atrophy	6	4
Progressive multifocal leukoencephalopathy	1	1
Diffuse cerebral edema	2	-
Superior sagittal sinus thrombosis	1	-
Ischemic lacuna	3	2
Basal ganglia vasculitis	-	1
Aspecific hypodensities	4	-
Aspecific hyperintense T2 signals	-	2

a With evidence of subdural collection.

b Some patients had more than one lesion.

HAART intake at baseline was not associated with any specific radiological pattern (data not shown). Based on 59 CT images, there was no difference in the percentage of abnormal lesions as a whole, nor specifically of cryptococcosis-related lesions according to the infecting serotype A (n = 49) or D (n = 10) (data not shown).

#### Relationship between baseline cerebral images and initial severity or outcome

We then assessed whether any of the radiological features were associated with initial severity or specific outcome. High serum and CSF antigens titers at baseline were recorded in 21/27 (78%) and 21/26 (81%) patients with cryptococcosis-related lesions respectively, but only in 13/31 (42%) and 14/28 (50%) patients without (p = 0.008 and p = 0.024, respectively). In the multivariate analysis, parameters independently associated with cryptococcosis-related cerebral lesions were high serum antigen titers (OR [95% CI] = 4.6 [1.4–15.1], p = 0.012) and neurological abnormalities (OR [95% CI] = 2.9 [0.9–9], p = 0.076). Similarly, high CSF antigen titers were associated with cryptococcosis-related lesions independently of neurological abnormalities (adjusted-OR [95% CI] = 4.3 [1.2–15.0], p = 0.02). A higher proportion of dilated VR spaces was found in patients with high serum antigen titers compared to patients without (11/34 (32%) vs. 2/24 (8%), p = 0.053). No association was found between the existence of cryptococcosis-related lesions and other markers of initial severity, i.e. low CSF white-cell count, elevated CSF protein concentration or hyponatremia at baseline (data not shown).

No statistically significant association was found between cryptococcosis-related lesions as a whole or any specific lesions seen at baseline and the occurrence of mycological failure at the week 2 and month 3 workups or further development of IRIS. Indeed, the 4 patients with IRIS had various baseline CT lesions (intracerebral masses, aspecific *caudate nucleus* hypodensities, cortico-subcortical atrophy and no lesion, n = 1 each).

#### Comparison of CT and MR results for patients with dual exploration

Seventeen patients underwent dual exploration because of local protocols, or slightly delayed availability of MRI (Table 2). Clinical presentation for these patients was similar to those reported for the group of patients who had undergone a single radiological investigation, with the same proportion of reported neurological abnormalities (9/17 (53%) and 18/45 (40%) respectively, p>0.5). However they appeared to be more severely infected than the rest of the cohort, with a significantly higher frequency of elevated serum antigen titers (14/17 (82%) vs. 20/41 (49%) respectively, p = 0.022) and CSF antigen titers (14/16 (88%) vs. 21/38 (55%), respectively, p = 0.03). For these 17 patients, cryptococcosis-related lesions were significantly more frequently observed by MR than by CT (13/17(76%) vs. 4/17 (24%), p = 0.005), with more frequent visualization of abnormal VR spaces by MR than by CT (8/17 (47%) versus 1/17 (6%), p = 0.017). The proportion of other lesions, however, did not differ according to the investigation performed.

**Table 2:** Initial computed tomography and magnetic resonance findings among the 17 patients with dual explorations during culture-proven cryptococcal meningoencephalitis.

Imaging findings, % (n)	Computed tomography	Magnetic resonance	p-value
Normal	47.1% (8)	5.9% (1)	0.085
Cryptococcosis-related lesions	23.5% (4)	76.4% (13)	0.005
VR dilatation	5.9% (1)	47.1% (8)	0.017
Pseudocysts	0	11.8% (2)	0.485
Intracerebral nodule(s) or mass(es)	17.6% (3)	17.6% (3)	1
Hydrocephalus	0% (0)	0% (0)	-
Radiological meningitis	0% (0)	5.9% (1)	1

### Radiological findings during the course of cryptococcal meningoencephalitis

Clinical and mycological data were available for 57 patients during follow-up. Of these, 12 (21.5%) died before month 3, with death related to cryptococcosis for 7/12 (58%). Four patients were subsequently diagnosed with IRIS within a median of 11 months (range, 3–38 months) and neurological sequelae were reported in 6 of the 45 survivors (14%).

Of the 57 patients, 24 patients had repeated radiological procedures, allowing comparison of consecutive images over 3 months. At baseline, these 24 patients were more severely infected with more frequently higher serum antigen titers than the other patients (18/23 (78%) vs. 46%, p<0.001). They also had more subsequent neurological sequelae (5/16 (31%) vs. 1/28 (4%), p = 0.018).

No correlation was found between radiological evolution (exacerbation, stability or improvement of cryptococcosis-related or unrelated lesions) and outcome at month 3 including occurrence of IRIS.

### Review of reported series in the HIV-infected population

All published series were performed during the pre-HAART era and were retrospective in design (Table 3) [4]–[6], [26], [28]–[34]. They involved less than 20 patients in all but 4 studies [4], [7], [30]–[31]. Four of them involved the use of autopsy findings in order to ascertain any radio-pathological correlations. [5], [7], [26], [35]. Taking into account this semantic diversity, the main lesions visualized by CT in these pre-HAART series were masses (6 to 37.5%, [4], [5]), hydrocephalus (8 to 20%, [30], [34]) and pseudocysts (6 to 40%, [26], [31]), while the main lesions observed by MR were dilated VR spaces (56 to 100%, [5]–[6]), masses (11 to 60%, [4], [6]) and radiological meningitis (17 to 78%, [6], [29]), best visualised after double dose contrast medium injection [6].

**Table 3:** Published series (1982–2007) of more than 5 HIV-infected adult cases on radiological presentation of cerebral cryptococcosis.

References	Year	Country	HIV-status	No. of patients w CT^a^ or MR^b^	Main findings, n (%)						
					Normal	Dilated VR spaces	Pseudocysts	Mass(es)	Hydroce phalus	Radiological meningitis	Other lesions
[28]	1985	USA	HIV+	10 CT	8 (80%)	-	-	-	-	-	2 (20%)
[32]	1986	USA	HIV+	13 CT	9 (69%)	-	-	2 (15%)	1 (8%)	-	1 (8%)
[31]	1990	USA	28 HIV+	35 CT	15 (29%)	-	2 (6%)	2 (6%)	3 (9%)	-	13 (37%)
			7 ID HIV−								
[4]	1990	USA	HIV+	29 CT	9 (31%)	-	-	5 (17%)	-	-	15 (52%)
[4]	1990	USA	HIV+	10 MR*	0	6 (60%)	-	6 (60%)	-	-	-
[5]	1992	USA	HIV+	8 CT	1 (12.5%)	-	-	3 (37.5%)	-	-	3 (37.5%)
[5]	1992	USA	HIV+	5 MR	0	5 (100%)	-	1 (20%)	-	1 (20%)	2 (40%)
[6]	1993	Italy	HIV+	9 MR	0	5 (56%)	1 (11%)	1 (11%)	-	7 (78%)	-
[29]	1994	Germany	HIV+	8 CT	5 (62.5%)	-	-	-	-	-	3 (37.5%)
[29]	1994	Germany	HIV+	6 MR	3 (50%)	-	-	1 (17%)	-	1 (17%)	1 (17%)
[30]	1996	France	HIV+	48 CT	33 (69%)	-	-	4 (8%)	4 (8%)	2 (4%)	5 (10%)
[33]	1997	Spain	HIV+	17 CT	8 (41%)	-	-	2 (12%)	-	-	8 (47%)
[34]	1997	South Africa	HIV+	15 CT	12 (80%)	-	-	-	-	-	3 (20%)
[26]	1997	USA	HIV+	10 CT	4 (40%)	-	4 (40%)	-	2 (20%)	-	-
Our study	2007	France	HIV+	55 CT	26 (47%)	3 (5%)	2 (4%)	5 (9%)	2 (4%)	2 (4%)	20 (36%)
Our study	2007	France	HIV+	24 MR	2 (8%)	11 (46%)	2 (8%)	5 (21%)	-	2 (8%)	11 (46%)

a CT: computed tomography.

b MR: magnetic resonance image.

Radio-pathological comparisons underlined the overall low performance of both MR and CT to detect fungal meningitis which was a constant at autopsy [5], [26], [36]. Several studies pointed out the lack of radiological hydrocephalus in cases of high opening CSF pressure [21], [29], [37]–[40]. Radiological cerebral atrophy was frequently noted (≥30% [4], [31]). CT images were reported as normal in more than 40% of cases in the 10/12 series and MRI reported as normal in around 10% of cases in the 4 MR pre-HAART series.

## Discussion

We analysed the radiological features of cerebral cryptococcosis in a large prospective cohort of HIV-infected patients in the HAART era and compared brain images with parameters assessing disease severity [3].

The pattern of cryptococcosis-related lesions recorded by CT consisted, here, of predominantly dilated VR spaces, masses and meningitis and on MR images, of mass(es) and pseudocysts with, rarely, radiological meningitis and hydrocephalus, as reported in the pre-HAART era in the available series (Table 3) [4]–[6], [26], [28]–[34]. Radiological cerebral atrophy however appeared less frequently here than previously reported (≤13% here vs. ≥30% [4], [31]), probably because of the diminished incidence of HIV-associated dementia and because cryptococcosis is more often revelatory of AIDS than in the pre-HAART era [41]. Thus, it would appear that the advent of HAART has had no influence on the development of cryptococcal-related lesions (including inflammatory lesions) despite its demonstrated effect on fungal virulence and on the local production of IL-8 [9]–[10], [42]. Strikingly, and in agreement with pre-HAART studies, normal brain imaging (47% by CT and 8% by MR) did not rule out cryptococcal meningoencephalitis. Finally, as reported elsewhere [43], other opportunistic infections can be concomitantly diagnosed (as was the case in 2 of our patients).

The value of brain imaging for assessing AIDS-associated cryptococcosis' initial severity was analyzed. We have previously shown that abnormal brain images were associated with a higher risk of death within three months after the diagnosis [3]. Here, a significant association was found between the existence of cryptococcosis-related radiological brain lesions at baseline and high serum or CSF antigen titers, two major prognostic markers [3]. This association was found even in patients with normal neurological examinations. The Infectious Diseases Society of America guidelines for the management of cryptococcosis, published in 2000, recommend performing neuroimaging prior to lumbar puncture in cases of neurological abnormalities [44]. Our study suggests that: (i) neuroimaging, especially MR, should be considered as a valuable tool to assess cryptococcosis' initial severity in HIV-infected patients, along with other prognostic markers and that (ii) cryptococcosis-related lesions may deserve appraisal even in the absence of neurological abnormalities if a high fungal burden is suspected on the basis of high serum and/or CSF antigen titers.

The question of which investigation should be performed is often answered by which is available faster. MR has clearly been demonstrated to be more accurate than CT for the investigation of other cerebral lesions [29], [45]–[46]. Two preliminary comparisons of CT and MR in,respectively, 10 and 8 immunocompetent/compromised patients with cryptococcal meningoencephalitis suggested a higher efficiency of MR over CT for the visualization of VR spaces [4], [47] possibly because of the limited inflammation [5], [26]. Here, cryptococcosis-related lesions were significantly more frequently observed on MR than on CT images for 62 HIV-infected patients including 17 for whom both investigations were performed. Of note, the VR spaces which appear as the main anatomical site involved, radiologically, during cerebral cryptococcosis are also the site of brain invasion associated with fungemia in a relevant murine model of disseminated cryptococcosis [48]–[50]. Considering its high performance, cerebral MR imaging of infected mice should be a powerful tool for further dissection of cerebral cryptococcosis pathophysiology.

The current guidelines do not comment on the need to repeat radiological investigations [44]. Twenty four patients in our cohort had multiple neuroimaging. They were more likely to have more severe disease and a poorer outcome, an observation consistent with daily practice. Our data from this large subgroup of patients does not support repeated neuromaging in clinically and mycologically stable patients. However, neurologically unstable patients would benefit from further radiological evaluations, keeping in mind the possibility of new opportunistic infections and IRIS [51]–[53].

In conclusion, our study suggests that brain imaging, especially by MR, is an additional effective tool in the assessment of initial disease severity in AIDS-associated cryptococcosis. The absence of neurological abnormality should not preclude neuroimaging especially in cases of suspected high fungal burden on the basis of high antigen titers.

## Materials and Methods

### Study population

Sixty two patients with culture-proven cryptococcal meningoencephalitis and available brain imaging were analysed from 21 hospitals in Paris area. These patients were enrolled onto the nationwide multicentric prospective CryptoA/D study [3]. Written informed consent had been obtained in accordance with the French Ethical Committee Recommendations.

Clinical, biological and mycological data were available at baseline for all patients and at 2 weeks and 3 months of antifungal therapy for 57 patients. Initial severity and prognosis was evaluated, as already described [3]. Briefly, initial severity was assessed according to the existence of neurological abnormalities and high (≥512) serum or CSF cryptococcal antigen titers. Low CSF white-cell count (<20/µl), elevated CSF protein concentration or hyponatremia were also recorded. Mycological failure after 2 weeks of antifungal therapy corresponded to the persistence of viable yeasts in at least one body system. Treatment failure was defined for the patients followed up to 3 months after the diagnosis of cryptococcosis and consisted of death, persistence of viable yeasts in culture or neurological sequelae. Immune reconstitution inflammatory syndrome (IRIS) was defined according to the literature [52].

### Radiological investigations

Neuroimaging was performed according to local practices. All brain CT and MR images available from baseline to 3 months after the diagnosis were collected and blindly analyzed by neuroradiologists experienced in the field of AIDS-associated opportunistic central nervous system infections. Brain lesions were recorded following a pre-established checklist.

The following items were analysed according to the available pre-HAART literature on brain cryptococcal lesions: dilated Virchow-Robin (VR) spaces [4]–[7], [26], [54], pseudocysts (also called “gelatinous pseudocysts” [6] and “soap bubbles” [35], corresponding to fungal proliferation in VR spaces invading the surrounding parenchyma [6] or to confluent VR spaces [26]); intracerebral nodules or masses (also called nodular lesions [4], granulomas [55], cryptococcomas [5]–[7], [31] or brain fungal abscesses [31]–[32], [56]–[57]); hydrocephalus and radiological meningitis or its complications [58]. Cryptococcosis-related lesions were, here, defined by the presence of at least one of the following images: dilated VR spaces, pseudocysts, intracerebral masses and hydrocephalus. Images of progressive multifocal leukoencephalopathy, cerebral atrophy, or features compatible with HIV encephalopathy were also noted, as well as any other abnormalities. Lesions were considered as sequelae if they had the appearance of ischemic infarcts, were calcified without peripheral edema or mass effect and did not enhance with intravenous contrast enhancement, and were thus excluded from the analysis.

Baseline MR and CT images were obtained within 7 days before and up to 14 days after the mycological diagnosis of cryptococcal meningoencephalitis. A minimum of 2 weeks was required between two consecutive procedures to evaluate potential changes. Dual exploration (CT and MR for the same patient) was defined if both investigations were performed within a period of two consecutive weeks. Radiological evolution was evaluated on images obtained by the same procedure (CT or MR) and classified as an exacerbation (i.e., increase in size and/or number of lesions or apparition of any new lesions), a complete or partial response (i.e., disappearance or decrease of more than 50% in the number and/or size of lesions), or otherwise as stable.

### Review of reported series in the HIV-infected population

We reviewed and analyzed the literature through a Medline search (up to October 2007). All series including more than 5 HIV-infected cases published in English or French were selected using combinations of brain/cerebral, magnetic resonance imaging/computed tomography, X-Ray, and cryptococcosis/cryptococcal, meningitis/meningoencephalitis as key words. Cases due to *C. gattii* were excluded because of the reported distinct pattern in terms of geographical distribution, underlying diseases and types of tissue lesions [59].

### Statistical analysis

The prevalence of every radiological feature at baseline was determined for all the CT and MR images collected. Then, the association between the presence of cryptococcosis-related lesions at baseline and initial severity parameters with the subsequent occurrence of mycological failure, death, neurological sequelae or IRIS was studied using results obtained with either CT or MR (and considering only MR results in cases of dual exploration).

Statistical analysis was performed by using the STATA 8.0 statistical package (Stata Corporation, College Station, Texas, USA). Continuous variables were compared using the non-parametric Mann-Whitney rank sum test. The chi-square or Fisher's exact test was used to assess significant relationships between discrete variables. The Mac Nemar exact test was used to compare the proportion of positive findings with CT and MR in cases of dual exploration. Two-sided p values less than 0.05 were regarded as significant. Multivariate analysis with logistic regression was performed to determine factors associated with cryptococcosis-related lesions. Odds ratios (OR) and their 95% confidence intervals [95%CI] were determined by means of logistic regression analysis. All variables that were clinically relevant with p value <0.25 in univariate analysis were entered simultaneously into the initial model. A backward stepwise procedure was used to remove variables until all variables retained in the final model had p values <0.05. Interactions were explored by means of interaction terms added to the logistic regression model.
